# Visualizing Cancer Cell Metabolic Dynamics Regulated With Aromatic Amino Acids Using DO-SRS and 2PEF Microscopy

**DOI:** 10.3389/fmolb.2021.779702

**Published:** 2021-12-15

**Authors:** Pegah Bagheri, Khang Hoang, Anthony A. Fung, Sahran Hussain, Lingyan Shi

**Affiliations:** Department of Bioengineering, University of California San Diego, San Diego, CA, United States

**Keywords:** SRS, aromatic amino acids, metabolism, metabolic dynamics, phenylalanin, tryptophan, DO-SRS, lipid metabolism

## Abstract

Oxidative imbalance plays an essential role in the progression of many diseases that include cancer and neurodegenerative diseases. Aromatic amino acids (AAA) such as phenylalanine and tryptophan have the capability of escalating oxidative stress because of their involvement in the production of Reactive Oxygen Species (ROS)_._ Here, we use D_2_O (heavy water) probed stimulated Raman scattering microscopy (DO-SRS) and two Photon Excitation Fluorescence (2PEF) microscopy as a multimodal imaging approach to visualize metabolic changes in HeLa cells under excess AAA such as phenylalanine or trytophan in culture media. The cellular spatial distribution of *de novo* lipogenesis, new protein synthesis, NADH, Flavin, unsaturated lipids, and saturated lipids were all imaged and quantified in this experiment. Our studies reveal ∼10% increase in *de novo* lipogenesis and the ratio of NADH to flavin, and ∼50% increase of the ratio of unsaturated lipids to saturated lipid in cells treated with excess phenylalanine or trytophan. In contrast, these cells exhibited a decrease in the protein synthesis rate by ∼10% under these AAA treatments. The cellular metabolic activities of these biomolecules are indicators of elevated oxidative stress and mitochondrial dysfunction. Furthermore, 3D reconstruction images of lipid droplets were acquired and quantified to observe their spatial distribution around cells’ nuceli under different AAA culture media. We observed a higher number of lipid droplets in excess AAA conditions. Our study showcases that DO-SRS imaging can be used to quantitatively study how excess AAA regulates metabolic activities of cells with subcellular resolution *in situ*.

## Introduction

**FIGURE 1:**
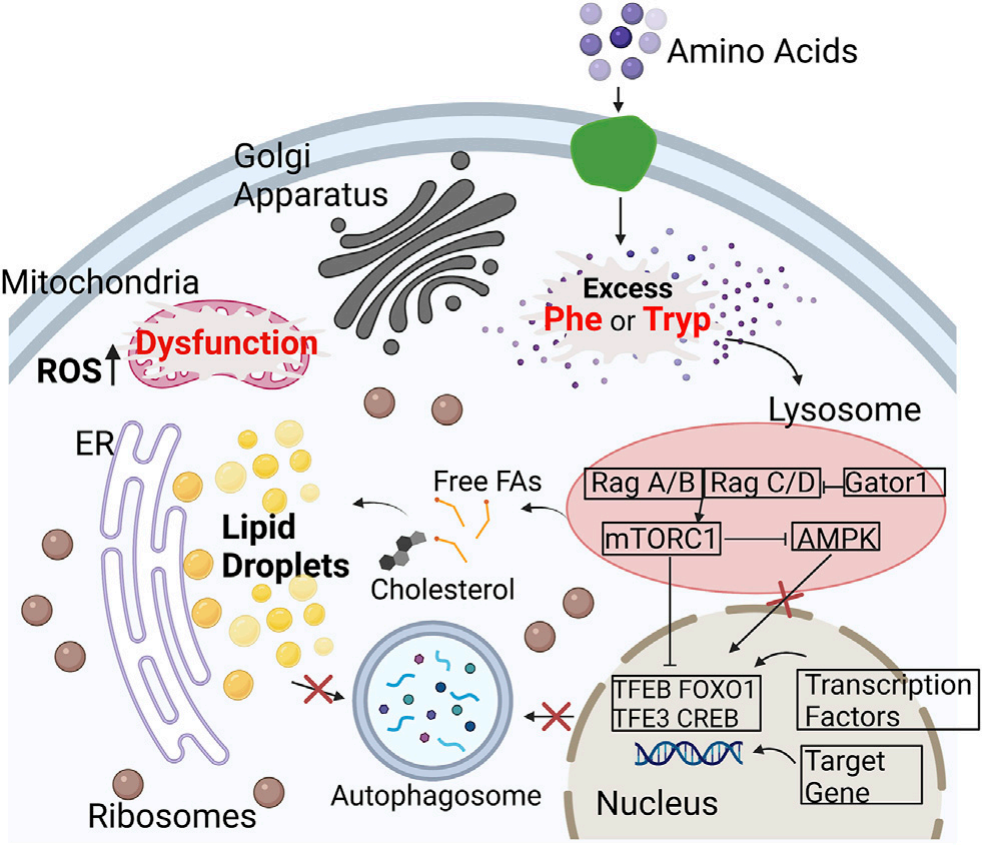
Illustration of how excess amino acids upregulate the mTORC1 pathway in cells. mTOR is a key regulator of lipid metabolism. With higher available nutrients, such as Phe (Phenylalanine) and Tryp (Tryptophan), mTORC1 is active and AMPK becomes inactive. mTOR inhibits the transcription factors, that include TFEB and TFE3, to block the targeting of transcriptional genes associated with the autophagy-lysosome (Chiacchiera and Simone, 2009; Thiem et al., 2013; Vadlakonda et al., 2013; Zhao et al., 2017). This ultimately results in lack of autophagy and start of lipophagy leading to an increase in lipid droplet accumulation and ROS. The increase in oxidative stress results in the dysfunction of the mitochondria and ER stress (Zhang et al., 2018; Paquette et al., 2021; Ralhan et al., 2021). Abbreviations: (AMPK), mechanistic target of Rapamycin complex 1 (mTORC1), (TFEB), (TFE3).

The aromatic amino acids (AAA), L-phenylalanine and L-tryptophan, are essential for protein synthesis (Parthasarathy et al., 2018), and serve as functional components in the regulation of many metabolic pathways (Wu, 2013) with implications in diseases such as cancer (Kimura and Watanable, 2016; Cheng et al., 2019; Ma et al., 2021). AAAs can be critical intermediates that connect nucleotide, glucose, and lipid metabolism (Wei et al., 2020), but may also serve as energy sources for proliferating cancer cells. Oxidative imbalance and stress also play essential roles in the progression of cancer (Moneim, 2015; Saha et al., 2017). For instance, increased oxidative stress is a hallmark of the aging process, and continued oxidative stress can induce chronic inflammation that leads to cancer (Reuter et al., 2010). Therefore, the regulation of AAAs has the potential to amplify oxidative stress during the onset and progression of diseases. This is because excess AAA, such as phenylalanine and tryptophan, can induce the production of Reactive Oxygen Species (ROS) by activating the mammalian target of rapamycin (mTOR) and promoting oxygen consumption and mitochondrial metabolism (Wang et al., 2015; Saxton and Sabatini, 2017a; Mossmann et al., 2018). In addition, mechanistic target of rapamycin complex 1 (mTORC1) becomes specifically activated with excess AAA while the AMP-activated protein kinase (AMPK) is inhibited (Chiacchiera and Simone, 2009; Thiem et al., 2013; Vadlakonda et al., 2013; Zhao et al., 2017). Cell growth and metabolism rely on mTORC1 as a critical regulator through the modulation of lipid and protein synthesis, autophagy, and biogenesis (Takahara et al., 2020). Dysregulation of mTORC1 and related enzymes such as AMPK is associated with diseases such as cancer and neurodegenerative disorders (Blommaart et al., 1995; Sengupta et al., 2010; Saxton and Sabatini, 2017b; Takahara et al., 2020). AMPK maintains the production and consumption of ATP in eukaryotic cells (Rossi et al., 2018) and is critical for the activity of transcription factors TFEB and TFE3 (Hardie, 2007). Its inhibition blocks the targeting gene associated with autophagy-lysosome and accounts for the accumulation of lipid species (Figure 1). With the failure of autophagy and lipophagy initiation, an accumulation of lipid droplets (LDs) and increase in ROS within cells can perhaps be observed and quantified. Ultimately, these altered metabolic activities can contribute to mitochondrial dysfunctions that lead to the production of malignant precursors from healthy cells (Porporato et al., 2018). Furthermore, LDs can reflect cellular stress as oxidative stress can lead to LD accumulation (Khatchadourian et al., 2012; Younce and Kolattukudy, 2012; Lee et al., 2013). Dysfunctional mitochondria are indicative of cancer progression (de la Cruz López et al., 2019), and the upregulation of mTORC1 is implicated in the lipid metabolism of mitochondria. Due to the lack of non-invasive, label-free imaging methods, the role of AAA, specfically phenylalanine and trytophan, in cellular metabolism such as lipid synthesis and protein synthesis is unclear.

AAA studies usually rely on gas chromatography (GC) and/or mass spectroscopy (MS)-based imaging techniques to study lipids (Li et al., 2021). Electrospray ionization (ESI)-MS has also been used to study how fatty acids quantitively change with AAA supplementation (Ma et al., 2021); however, these imaging technologies lack the ability to show the lipids’ spatial distribution in cells. Other methods, such fluorescence microscopy or magnetic resonance imaging (MRI), require fluorescent dyes or simply have limited spatial resolution. Moreover, the required dyes for some of these techniques can potentially interfere with molecular activities happening within the cells (Gialleonardo et al., 2016). On the other hand, matrix-assisted laser desorption/ionization (MALDI) has been made used to study how biomarkers can regulate fatty acid metabolism in cancer cells without affecting native distributions (Pirman et al., 2013), but suffers from relatively shallow imaging depths, and poorer spatial resolution despite the additional sample preparation (Murphy et al., 2009; Bowman et al., 2020). Atomic force microscopy is another powerful technique that can be used to observe lipid formation; however, it is difficult to study the miscibility of multiple lipids (Wang et al., 2012).

**FIGURE 2:**
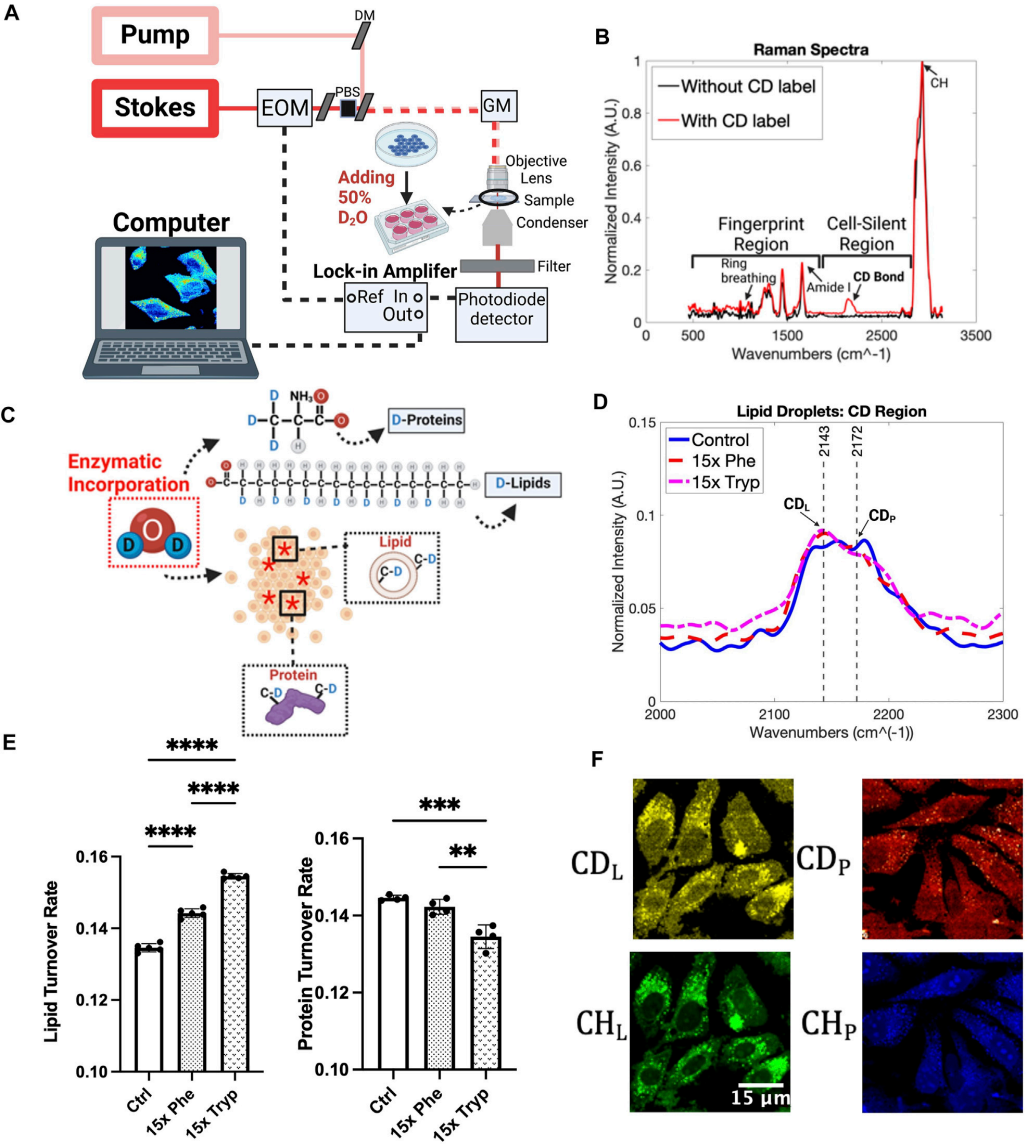
DO-SRS microscopy and Spontaneous Raman spectra. **(A)** Schematic diagram of the experimental setup SRS system workflow (Li et al., 2017). Abbreviations: DM (dichroic mirror), EOM (electro-optical modulator), PBS (polarizing beam splitter), GM (galvometer mirror). **(B)** Spontaneous Raman spectra without D_2_O incorporation (black) and 50% of D_2_O addition (red). **(C)** Magnification of how HeLa cells were treated with D_2_O and excess aromatic amino acids, Phenylalanine (Phe) and Tryptophan (Tryp). Red asterisks represent the effect of D_2_O to the cells. **(D)** Results from processed Spontaneous Raman spectrums for the control (Ctrl) group (blue), 15x Phenylalanine-treated (15x Phe) group (red), and 15x Tryptophan-treated (15x Tryp) group (magenta) from HeLa cells. Spectrums were taken with the laser focused on structures that resembled lipid droplet organelles under the brightfield imaging system. Each Raman spectrum had a background spectrum from the PBS removed before being normalized, averaged, and baseline corrected. Peaks labeled correspond to Deuterium-labelled lipid (CD_L_) and Deuterium-labelled protein (CD_P_) (Yamakoshi et al., 2011; Shi et al., 2018a; Shi et al., 2018b; Zhang et al., 2019; Zhao et al., 2020), which were observed more specifically to understand how 50% D_2_O cell media with the excess aromatic amino acids affected the lipid metabolic activity. **(E)** Quantification of the mean Raman results from CD_L_ and CD_P_ peaks for HeLa under control and experimental conditions. ****p < 0.0001, ***p < 0.001, **p < 0.01 from 2 way ANOVA test. **(F)** DO-SRS images of HeLa cells in C-D lipid (2145 cm^−1^) and C-D protein (2175 cm^−1^) on the top row. Bottom row shows SRS images of cells in the protein and lipid channels from 2940 cm^−1^ and 2845 cm^−1^.

Raman spectroscopy and microscopy are relatively new optical techniques that are rapidly outpacing other molecule-specific imaging methods, and excel in high resolution and chemical specificity outputs in biological samples (Ghita et al., 2012; Daudon, 2016; Ember et al., 2017). This study makes use of Deuteriun-Oxide Stimulated Raman Scattering (DO-SRS) for the subcellular analysis of the AAAs regulated metabolic dynamics for molecular signatures including newly synthesized proteins and lipids non-invasively with minimal sample preparations. Heavy water was added to the cell culture media because the deuterium could be enzymatically included into proteins and lipids through *de novo* biosynthesis. Therefore, metabolic activities can be closely observed using this technique coupled with DO-SRS microscopy (Figure 2A). Figure 2B displays a typical Raman spectrum that can be obtained by our Raman spectroscopy using D_2_O media. Thousands of variables and their multiplexed patterns can be analyzed using a spectral resolution of 1.2 cm^−1^ and a range from 400 cm^−1^ to 3200 cm^−1^. The Raman peaks that display the strongest patterns, in terms of different intensities and positions, are then selected to be imaged with the DO-SRS microscopy to visualize the spatial distributions of these molecules within the cell. The output of these techniques is a hyperspectral image (HSI), where an optical focus plane is captured at different Raman shifts. The slice for the HSI displays the areas in which the specific molecular bonds exist, and pixel intensities are directly proportional to these molecular bonds’ concentrations (Shi et al., 2018a). Furthermore, Two Photon Excited Fluorescence (2PEF) microscopy is coupled with DO-SRS to provide additional information of flavin and nicotinamide adenine dinucleotide (NADH) pools in the same region of interest. Flavin and NADH autofluorescence have been associated with redox homeostasis in cells and can imply lipid peroxidation status as well (Mayevsky and Barbiro-Michaely, 2009; Surre et al., 2018). Thus, using both DO-SRS and 2PEF methods will allow us to visualize the metabolic dynamics of cells when AAAs are being regulated.

In this study, we utilized DO-SRS microscopy coupled with 2PEF microscopy to observe the metabolic activities of lipids and proteins in cancer cells and investigate the effects of AAA on LD metabolism in HeLa cells. Quantitative lipid synthesis rates of different experimental conditions help illuminate how lipid metabolism can be affected with the regulation of phenylalanine and tryptophan. The outcomes support AAAs as targets for the accumulation of LDs and ROS.

## Results

### Raman and DO-SRS Imaging to Identify Changes in Lipid and Protein Synthesis

Metabolic precursors can be tagged with deuterium before getting integrated into newly synthesized lipids and proteins (Miyagi and Kasumov, 2016), as shown in Figure 2C. DO-SRS enables one to visualize the subcellular spatial distribution of newly synthesized macromolecules with C-D bonds which have a distinguishable Raman peak in the cell silent region at 2150 cm^−1^ (Yamakoshi et al., 2011; Shi et al., 2018a; Shi et al., 2018b; Zhang et al., 2019; Zhao et al., 2020). 50% D_2_O in cell culture media produced distinct C-D Raman bands (Supplementary Figures S1A, S2B). Adding excess essential amino acids to our cell culture media at a 15x concentration introduced differences in the C-D band (Figure 2D). Indeed, with LDs being specifically observed, we noticed that both 15x phenylalanine and 15x tryptophan show higher intensities of biomolecules compared to the control group at the 2143 cm^−1^ peak. The lipid turnover rate was visualized with DO-SRS imaging and confirmed with spontaneous Raman spectra by showing that both aromatic amino acids have significant differences between each other and between the control group (Supplementary Figure S2B). Quantitative analysis can aid in understanding the subcellular resolution of LDs by calculating the ratios of the C-D lipid and protein peaks to their respective lipid (2850 cm^−1^) and protein (2930 cm^−1^) channels. Ten spectra were measured using Spontaneous Raman spectroscopy. The data was processed using MATLAB to subtract the background and conduct baseline correction. Resulting spectra were then vector normalized and averaged for each group. We observed a significant 7% and 15% increase in the lipid turnover rate between the control and 15x phenylalanine and 15x tryptophan case, respectively. This indicates that excess AAA has the potential to increase LD production. Interestingly, the protein in the C-D region, showed very minimal differences between the control and two experimental conditions (Figure 2D). The protein turnover rate, on the other hand, demonstrated an opposite trend to the lipid turnover rate. Specifically, there is a 1.38% decrease in the protein turnover rate between the control and 15x phenylalanine group, and a 6.6% decrease between the control and 15x tryptophan case (Figure 2E). However, the C-D protein (2185 cm^−1^), the protein channel (2930 cm^−1^), the C-D lipid (2150 cm^−1^) and the lipid channel (2850 cm^−1^) are just parts of the Spontaneous Raman spectra. Principal component analysis (PCA) shows that 19 principal components (PCs) account for 98% of the variance in the experimental groups of this study. A t-SNE diagram is used to visualize the top 15 principal components as shown in Supplementary Figure S1B. At least one dimension that distinguishes the effects of AAA on Raman spectra of HeLa cells under different treatments can be observed. This can show that even at 15x increase in concentration, we can still observe some profound differences compared to the control group. Although this verifies that phenylalanine and tryptophan do have a notable effect on lipid metabolism in cancer cells, PCA and t-SNE plots have limited ability to isolate specific peaks that contribute to major variances on Raman spectra in this study.

As various fluctuations of C-D protein and lipid signals were observed from Spontaneous Raman spectra of HeLa LDs, DO-SRS microscopy was used to visualize spatial distribution of C-D signals to a greater depth. A workflow of DO-SRS is displayed in Figure 2A. DO-SRS affords the convenience of visualizing lipid and protein metabolism simultaneously (Shi et al., 2018a). Both the C-D lipid and C-D protein channel were utilized, and the signals were clearly different between the control and the AAA conditions (Figure 2F). Image analysis highlights the spatial distribution of the lipids and proteins, and the *de novo* synthesis of these compounds emphasizes how much the LDs were affected by the excess phenylalanine and tryptophan.

### The Effect of Excess AAA on Lipid Metabolic Pathways Determined With DO-SRS Techniques

**FIGURE 3:**
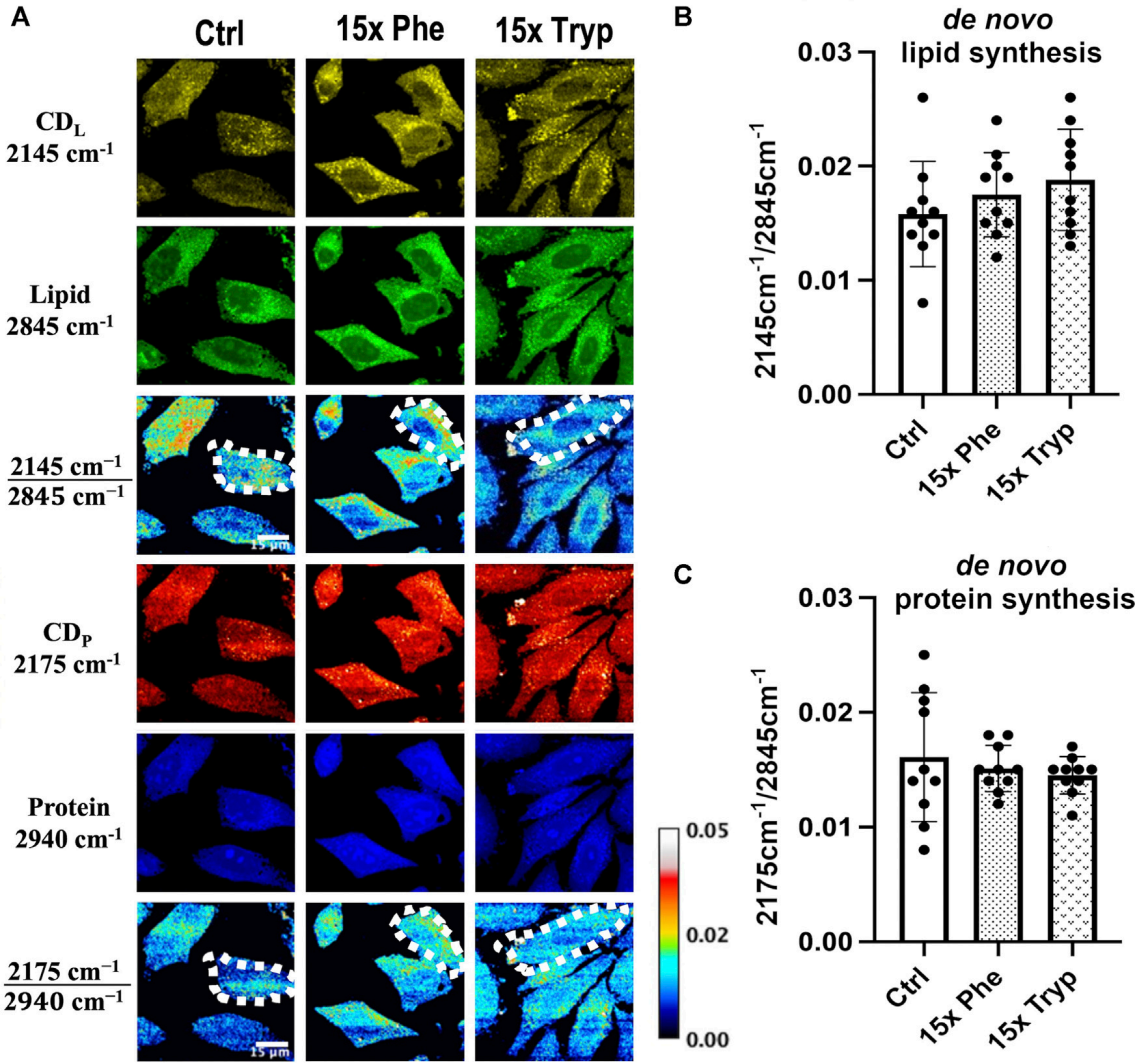
DO-SRS microscopy visualizes *in vitro* protein and lipid metabolism simultaneously in HeLa cells. **(A)** DO-SRS microscopy visualizes Deuterium-labelled lipid (CD_L_; 2145 cm^−1^), lipid (CH_2_; 2845 cm^−1^), Deuterium-labelled protein (CD_P_; 2175 cm^−1^) and protein (CH_3_; 2940 cm^−1^) channels in in HeLa cells for control (ctrl), 15x phenylalanine (15x phe), and 15x tryptophan (15x tryp). $\frac{2145\;cm-1}{2845\;cm-1}$ and $\frac{2175\;cm-1}{2940\;cm-1}\;$ ratios are calculated to understand metabolic activities of HeLa cells between the ctrl, 15x phe and 15x tryptophan 15x tryp groups. Multiple cell units are selected for calculating absolute intensities of $\frac{2145\;cm-1}{2845\;cm-1}$ and $\frac{2175\;cm-1}{2940\;cm-1}\;$ ratios between experimental groups. **(B,C)** Ratiometric analysis of lipid and protein turnover rates to compare control group with experimental conditions with p-value of 0.1498 and 0.6816, respectively from the 2-way ANOVA test.

DO-SRS imaging of experimental and control groups show differences between LD signals. Using ImageJ, the same amount of cell units from three samples are manually segmented and measured, as indicated by dotted-white borders in Figure 3A. With only the control case, there is some signal in the C-D lipid and protein channel; however, the 15x phenylalanine (15x Phe) and 15x tryptophan (15x Tryp) display even stronger signals (Figure 3A). The 15x Tryp case shows a profound signal for lipid droplets in the C-D lipid channel but a weaker signal in the C-D protein channel. A similar trend occurs for the 15x Phe as compared to the control case for both lipid and protein channels. Quantitative analysis shows the accumulation of synthesized lipid and protein (Figure 3B). Although the statistical analysis did not show significance, the ratiometric results demonstrate that the AAA conditions have greater lipid synthesis by 10–17% but reduced protein synthesis by approximately 10%. Protein synthesis is critical in the study of metabolic dynamics, and although D-labeled amino acids can be used to tack protein synthesis (Li et al., 2017), D_2_O has proven to be more efficient and consistent (Shi et al., 2018a). From our results, an increase in AAA can have the potential to increase lipid synthesis but decrease protein synthesis using 50% v/v D_2_O in the cell culture media. This evidence infers the possibility of a lack of autophagy taking place in the cells that causes an increase in LDs and promotes the generation of ROS as well as mitochondrial dysfunction (Chiacchiera and Simone, 2009; Younce and Kolattukudy, 2012; Nguyen et al., 2017; Zhang et al., 2018). DO-SRS has proven to be very effective and accurate to track the lipid and protein synthesis of HeLa cells and observing how AAA can show critical changes of the cells’ signal.

**FIGURE 4:**
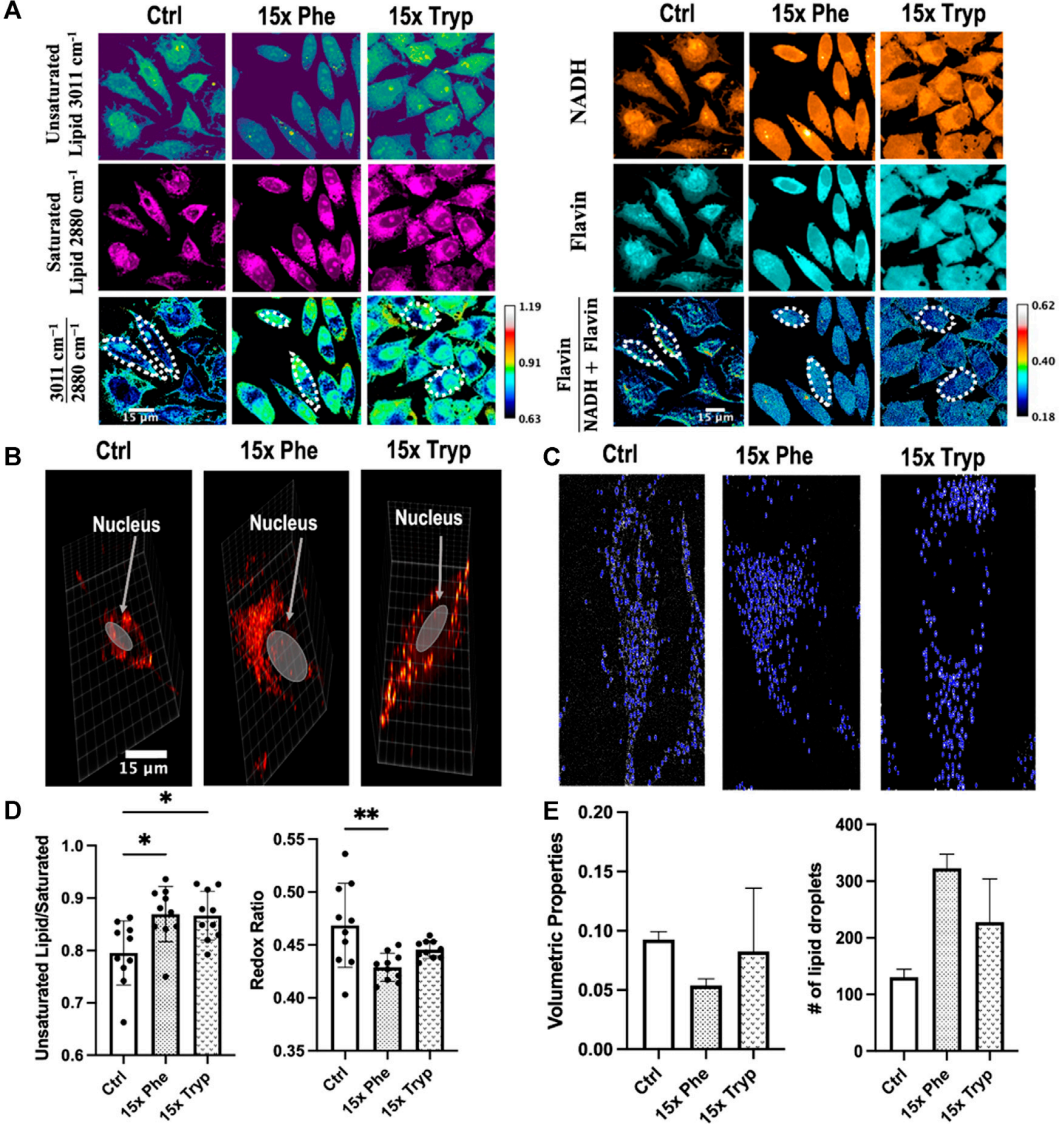
Images obtained using label-free SRS and analyzed from the unsaturated fatty acids, saturated fatty acids, NADH, and Flavin channel to observe the autofluorescence between each experimental condition. **(A)** SRS microscope visualizes *in vitro* unsaturated fatty acid (3011 cm−1) and saturated fatty acid (2880 cm−1) channels, and 2PEF microscope visualizes *in vitro* Flavin and NADH channels in HeLa cells for control (ctrl), 15x phenylalanine (15x phe), and 15x tryptophan (15x tryp). Many cell units in the same regions of interest are used for calculating absolute intensities of $\frac{3011\;cm-1}{2880\;cm-1}$ and $\frac{\mathrm{NADH}}{\mathrm{Flavin}}$ . **(B)** Single cell maximum intensity projection using SRS image stacks. LDs are highlighted in blue color using a home-made MATLAB script. **(C)** The volume and average number of LDs for control and experimental conditions. Excess AAA LDs have a reduced size compared to control group; however, they have a greater number of LDs. **(D,E)** Quantifications of $\frac{3011\;cm-1}{2880\;cm-1}$ and $\frac{\mathrm{NADH}}{\mathrm{Flavin}}$ concentration for HeLa cells under control and experimental conditions for given regions of interest. Excess amino acid treated groups shows large statistical significances compared to the control (ctrl) group. ****p < 0.0001, *p < 0.05 from 2-way ANOVA test. **(D)** SRS microscope visualizes the 3D spatial distribution of lipid droplets on a single cell unit in the control (Ctrl), 15x Phenylalanine (15x Phe) and 15x Tryptophan (15x Tryp) groups.

Using SRS and 2PEF microscopy, multimodality imaging of unsaturated lipid (∼3011 cm^−1^), saturated lipid (∼2880 cm^−1^) and flavin, NADH signals were acquired (Figure 4A). Similarly, the same amount of cell units from three samples are manually segmented and measured using ImageJ as indicated by the dotted-white borders in Figure 4A. Oxygen is a critical metabolite that accepts electrons from reduced NADH and flavin at the end of the electron transport chain (ETC) (van Manen et al., 2008). However, electrons can escape NADH and flavin before reaching the end of the ETC in mitochondria to produce ROS. In some cancer cells, the accumulation of ROS induces the oxidation of polyunsaturated fatty acids, promotion of saturated lipid production, and depletion of NADH levels (Watmough et al., 1990; Wang et al., 2019; Zhang and Boppart, 2021) in response to the oxidative stress. However, HeLa cells have been shown to increase polyunsaturated lipid levels in response to elevated ROS (Cruz et al., 2020). Raman shifts have been used to describe the magnitude of unsaturated and saturated lipids at 3011 cm^−1^ and 2880 cm^−1^ (Supplementary Figure S2A), respectively (Da Silva et al., 2009; Jamieson et al., 2018).

Ratiometric SRS images demonstrate that excess AAA-treated cells have an elevated ratio of unsaturated lipid/saturated lipid by 10% as compared to the control HeLa cells. However, neither the lipid saturation nor the optical redox ratio were significantly different between the two excess AAA-treated cells (Figure 4B). Quantitative analysis further showed statistical significance between the control group and the experimental groups for both the unsaturated lipid to saturated lipid and optical redox ratio, defined as Flavin/(Flavin + NADH) autofluorescence intensity. Moreover, the optical redox ratio demonstrated higher statistical importance between the two experimental groups with a 50% increase in the ratio (Figure 4D). The enrichment of NADH and flavin demonstrates an increase in the accumulation of LDs as β-oxidation is hampered (Watmough et al., 1990; Wang et al., 2019). Unsaturated lipid images were weaker than the saturated lipid channel which displayed a higher signal to noise ratio. With the manipulation of AAAs and the consequential changes in lipid content, LD structure, number, and distribution may be affected as well.

In addition to multimodal imaging, SRS has the capability to perform 3-D image reconstruction of LDs in control and AAA-treated HeLa samples. In short, the microscopy produces a set of cross-sectional images throughout the entire depth of a selected region of interest. In this study, we tune the stimulated Raman loss (SRL) to 2845 cm^−1^ and scan a region of interest from the top layer to the bottom layer with a step size of 1 micron (Figures 4B,C). Quantitative analyses of LD count and size were performed for each condition (Figure 4E). Both excess AAA-treated cells exhibited greater counts of LD but reduced volumetric properties compared to the control cell (Figure 4C). Qualitatively, the presence of excess Phe causes clusters of LD on one side of the cell’s nucleus, whereas the presence of excess Tryp leads to a uniform distribution of LDs around the cell’s nucleus (Figure 4B). During the span of this study, we were only able to investigate LD volume and counts of three single cell units per condition. Therefore, the results were not statistically significant, and require further research.

The abnormal accumulation or depletion of LDs are hallmarks and perhaps causes of various human pathologies (Thiam and Beller, 2017). LD-coating proteins greatly influence LD’s size and counts, ultimately affecting LD accumulation or depletion. For instance, Perilipins are a group of LD-coating with amphipathic helices (AHs) binding domains that target LD surfaces (Thiam and Dugail, 2019). Depending on the Perilipin species and their phosphorylated states, the binding of the proteins onto LD surfaces can initiate lipolysis or lipogenesis (Sztalryd and Brasaemle, 2017). Previous studies have shown that an increased number of bulky hydrophobic amino acid residues such as Tryp and Phe on the hydrophobic face of AH in these proteins can impair their binding affinity to LDs (Thiam and Dugail, 2019). It has been reported that with the failure of lipolysis from impaired Perilipins, there is an accumulation of numerous small LDs (Schott et al., 2019). Our 3D volumetric analyses of LDs concur with these previous findings by demonstrating that LDs increase in counts but decrease in volume in the excess AAA groups compared to the control group. Therefore, the presence of excess AAA might impair perilipin function and inhibit lipolysis, which can be visualized *in situ* with high resolution SRS.

## Discussion

In this study, we applied DO-SRS and 2PEF microscopies to investigate aromatic amino acids and their effects on redox homeostasis in HeLa cells. Lipid droplets were selected because they can provide critical metabolic insights for diseases such as cancer (Cruz et al., 2020) and heavy water was used to track newly synthesized lipid and protein. These carbon-deuterium bonds display distinct signals in the cell silent region on a Raman spectrum, allowing us to quantify and visualize these newly synthesized bonds *in situ*. In addition, 2PEF was used to provide spatial distribution of flavin and NADH at subcellular resolution. Therefore, by using DO-SRS and 2PEF in this study, we can understand metabolic changes of HeLa cells under different excess AAA treatments and advance current diagnostic methods for these diseases with our findings (Moneim, 2015; Parthasarathy et al., 2018).

Lipid, protein, flavin and other biomolecules have been proven to be indicators or metrics to measure progression of diseases (Heikal, 2010). With DO-SRS, we were able to visualize and quantify newly synthesized lipids and proteins. We observed increased *de novo* lipogenesis and a slight decrease in *de novo* protein synthesis in excess AAA treated groups compared to the control group (Figures 3B,C). These changes were supported by our Raman spectra collected by Spontaneous Raman Spectroscopy (Supplementary Figure S1). Hyperspectral images of unsaturated and saturated lipids were visualized at their respective wavenumbers to understand the effects of oxidative imbalance caused by excess AAA in HeLa cells. The ratios of unsaturated lipid/saturated lipid were calculated and compared across three conditions. The excess AAA-treated groups exhibited increased unsaturated lipid/saturated lipid compared to the control group. 2PEF was used to excite NADH and flavin in HeLa cells. The redox ratios of NADH/Flavin were calculated, compared, and related to the ratios of unsaturated lipid/saturated lipid. Both excess AAA-treated groups exhibited increased redox ratios compared to the control group. Previous studies have showcased that lower optical redox ratios correlated with increased ROS during cancer progression (Alhallak et al., 2016). In response to oxidative imbalance, HeLa cells display decreased levels of mono- and di-unsaturated, but increased levels of polyunsaturated lipids (Rysman et al., 2010; Munir et al., 2019). However, this effect varies across different cancer types (Lisec et al., 2019). In our study, both AAA-treated groups had elevated ROS by displaying lower optical redox ratios compared to the control. Because of the elevated ROS, both AAA-treated cells in our study supported previous findings by showcasing increased levels of unsaturated lipids and decreased levels of saturated lipids, ultimately leading to higher unsaturated lipids to saturated lipids ratios in AAA-treated cells compared to the control group.

In addition, 3D reconstruction images of LD were collected by our SRS system to study quantitative features such as counts and volume of LDs. Previous studies have demonstrated that LD-coating protein exhibit reduced binding affinity for LDs in the presence of excess AAA (Thiam and Dugail, 2019). Consequently, the failure of LD-coating protein binding can interfere with lipolysis and result in an accumulation of numerous small LDs (Schott et al., 2019). Our results support these studies by highlighting that both excess AAA groups exhibited lower volumetric properties, but higher counts of LDs compared to the control group. This outcome may infer the failure of binding of LD-coating protein onto LD surfaces and higher ROS synthesis rate (Moneim, 2015; Saha et al., 2017). However, further research to investigate is needed to confirm this finding using optical techniques.

In our study, excess AAA decreased the volume of LDs but increased their counts. Perhaps, this was done by affecting the binding affinity of LD-coating proteins onto LD surfaces and decreasing lipolysis. However, further investigation of lipid-protein interactions is needed to confirm DO-SRS data. Furthermore, lower optical redox ratios were observed in excess AAA-treated cells, which corrolated to elevated ROS and increased unsaturated lipids to saturated lipids ratios. To guard against oxidative stress and apoptosis induced by ROS, HeLa cells produce more unsaturated lipids than saturated lipids during their progression (Rysman et al., 2010; Munir et al., 2019). Therefore, our study provides a better understanding of imbalanced oxidative stress effects onto HeLa cells under excess AAA treatments and showcases DO-SRS coupled with 2PEF as non-invasive, high-resolution imaging systems to study metabolic activities *in situ*.

## Conclusion

In summary, this study showcases the effects of excess AAAs on cellular metabolic activities of cervical cancer cell lines and how lipid droplet phenotypes can be used as potential indicators in developments of future diagnostic methods for cancer and other closely related diseases. Without the need of labeling dyes that can interfere with normal physiological environments in a cellular sample, the state-of-the-art, non-invasive DO-SRS microscopy and 2PEF microscopy can visualize and quantify metabolic changes of various biomolecules including protein, lipid, flavin, NADH. In addition, Deuterium Oxide (D_2_O) allows us to locate these molecules *in situ* using C-D signals in the cell silent region with DO-SRS. In addition to phenylalanine and tryptophan, a water-insoluble tyrosine aromatic amino acid is not included in this study (Bowden et al., 2018). However, we intend to investigate the effects of tyrosine to obtain a holistic understanding of AAA on cellular metabolic activities in cancer and other closely related diseases. A non-linear optical second-harmonic generation (SHG) can also be applied to our SRS system to study collagen and their structure under the influence of AAA in HeLa xenograft models. Overall, our study demonstrates the useful applications of optical techniques such as DO-SRS and 2PEF in visualizing metabolic changes *in situ* with high resolution. Eventually, these methods can be translated to clinical settings to improve current diagnostic tools for diseases such as cancer and neurodegeneration.

## Materials and Methods

### Cell Culture

HeLa cells were cultured in Dulbecco’s modified Eagles’ medium (DMEM), supplemented with 10% fetal bovine serum (FBS) and 1% penicillin/streptomycin (Fisher Scientific, Waltham, MA), and incubated with 5% CO2 at 37°C. After passaging at 80% confluence, cells were seeded at a concentration of $2\times 10^{5}/\mathrm{mL}$ onto a 24-well plate. DMEM with 0.5% FBS and 1% penicillin/streptomycin was used to synchronize the cells for 8 h. The media was then changed to 50% (v/v) heavy water (D_2_O) and treatment media as described below.

For the excess aromatic amino acids condition, phenylalanine and tryptophan were increased as two separate test conditions at a 15x concentration. L-phenylalanine powder (SLCF3873, Sigma Aldrich) and L-tryptophan powder (SLCF2559, Sigma Aldrich) were added to DMEM for the excess groups. Cells were then incubated for 36 h and fixed on microscope slides afterwards. Next, the cells were gently rinsed with 1x PBS with Calcium and Magnesium ions at 37°C (Fisher Scientific, 14040216), and fixed in 4% methanol-free PFA solution (VWR, 15713-S) for 15 min. The cover glass was finally mounted on the cleaned 1mm thick glass microscope slides with 120 µm spacers filled with 1x PBS for imaging and spectroscopy. These samples are stored at 4°C when not in use.

### Spontaneous Raman Spectroscopy

A confocal Raman microscope (XploRA PLUS, Horiba) was used to obtain spontaneous Raman spectra. The microscope is equipped with a 532 nm diode laser source and 1800 lines/mm grating. The excitation power is approximately 40mW after passing through a 100x objective lens (MPLN100x, Olympus). The spectrometer collects the intensity values in each region for a range of excitation wavenumbers from 400 cm^−1^ to 3150 cm^−1^. The acquisition time used for these samples are 90 s with a binning of 4, and accumulation of 3 for minimal noise and greater accuracy for the resulting spectra (Supplementary Figure S1A) Each spectrum is taken by targeting the desired subcellular region and an additional spectrum is taken of the background with PBS in the same focal plane. Immediately after, the background spectrum is then subtracted from each subcellular target spectrum.

### Stimulated Raman Scattering Microscopy

An upright laser-scanning microscope (DIY multiphoton, Olympus) with a 25x water object (XLPLN, WMP2, 1.05 NA, Olympus) was used for near-IR throughput. Stokes with a wavelength at 1031 nm, 6 ps pulse width, and 80MHz repetition rate and synchronized pulsed pump beam with a tunable 720–990 nm wavelength, 5-6 ps pulse width, and 80 MHz repetition rate were supplied by the picoEmerald system (Applied Physics & Electronics) and coupled into the microscope. A high NA oil condenser (1.4 NA) was used for the collection of the Stokes and pump beams where the sample is mounted. For the water-immersion objective lens, a larger water droplet is placed on the glass cover slip of the sealed sample slide. The Stokes beam is blocked by a high O.D. shortpass filter (950 nm, Thorlabs) and transmits the pump beam onto a Si photodiode to detect the stimulated Raman loss signal. A lock-in amplifier at 20 MHz is utilized to terminate, filter, and demodulate the output current from the photodiode where the demodulated signal forms the image during the laser scanning as it is processed into the FV3000 software module FV-OSR (Olympus) as shown in Figure 2A. The images were collected at 512 × 512 pixels using a dwell time of 80 µs/pixel The images are saved as an OIR graphic file through the acquisition software by the Olympus microscope.

The background image was taken at 1900 cm^−1^ and subtracted from all the SRS images using ImageJ software. For multichannel SRS imaging, the pump wavelength $\left(\lambda_{\mathrm{pump}}\right)$ was tuned so that the energy difference between the pump and Stokes beams were matched with the vibrational frequency (Ralhan et al., 2021).

### Two Photon Excitation Microscopy

Label-free autofluorescence of flavins was excited at 800 nm and autofluorescence of NADH was excited at 780nm using the same tunable picosecond laser described in Stimulated Raman Scattering microscopy. Optical-parametric oscillators and amplifiers (OPO and OPA) sources provide tunable infra-red illumination. This takes place over a broad range of wavelength to allow for simultaneous multi-color imaging (Mahou et al., 2012). Back scattered emission of flavin and NADH autofluorescence was collected using a 460 nm/515nm filter cube (OCT-ET460/50M32, Olympus). These images were also 512 × 512 pixels and were acquired with an 8 µs/pixel dwell time using a 150mW power at the laser shutter.

### Data Analysis

#### Spectral Analysis

The mathematical modeling operations were conducted using MATLAB. Scripts and functions used for processing the Raman spectra were self-written using built-in functions provided by MATLAB. Origin software was used to display original spectra as shown in Supplementary Figure S1.

The spectral pre-processing consists of several steps that include background removal, baseline correction, and vector normalization. MATLAB software was used to import the raw spectra data. Background was subtracted, and the files were converted into an array where the spectra has been interpolated at every cm^−1^. The raw data was then graphed for verification, and the baseline correction was performed. The resulting spectra were vector normalized and averaged for each group to reduce the amount of noise on the graph of the biomolecular signals. Each spectral peak is assigned to the vibrations of a particular chemical bond or function group.

#### Image Analysis

Images were processed using MATLAB and ImageJ. To reduce horizontal noise artifacts caused by laser beam scanning, 3D image stacks of lipid droplets underwent bandpass filters and smoothing. Each lipid droplet received a spherical score by calculating the distance between its center of mass and surface. Subsequently, every spherical score of different lipid droplets were compared with a spherical score of a perfect sphere on the same Euclidean plan. Those lipid droplets with low sphericity scores were discarded. Using ImageJ, ten cell units from three different regions of interest per condition were manually segmented and measured. These cell units were relative in sizes, shapes and randomly selected for analysis.

#### Statistical Analysis

Statistical significance was verified by analysis of variance, checking the mean of more than two groups that are significantly different from each other. The mean and standard deviations were calculated for all the investigated conditions. The data were analyzed using GraphPad Prism for Mac. Significant differences between the controls and treatment groups were compared using the two-way analysis of variance (ANOVA). The data that had *p*-values lower than 0.05 were identified as statistically significant. MATLAB software was used to process the data for multivariate analysis. Additional statistical analysis was performed on Orange3 Data Mining Tool for greater visualization of the high-dimensional data such as primary component analysis (PCA) for more accurate representation of how the spectrums are compared from the control group to the excess aromatic amino acid groups.

## Data Availability

The raw data supporting the conclusion of this article will be made available by the authors, without undue reservation.

## Supplementary Material

Supplementary Figure S1 Spectra obtained from Spontaneous Raman spectroscopy using 90 second acquisition time and accumulation of 3. Analysis of Spontaneous Raman spectroscopy results from HeLa cells. **(A)** Results obtained from control and experimental conditions: 15x Phenylalanine and 15x Tryptophan. **(B)** t-SNE distributions between control treated samples and excess AAA treated samples.

Supplementary Figure S2 Multiplexed imaging of HeLa cells between control (ctrl), 15x Phenylalanine (15x Phe), and 15x Tryptophan (15x Tryp) using label-free SRS and DO-SRS imaging systems. **(A)** Multiplexed images of ctrl, 15x Phe, and 15x Tryp of protein (CH_3_; 2940 cm^−1^), lipid (CH_2_; 2845 cm^−1^), unsaturated lipid (3011 cm^−1^), saturated lipid (2880 cm^−1^) and 1740 cm^−1^ channels. Hematoxylin and Eosin staining images are produced by combining the protein and lipid channels using FIJI. **(B)** DO-SRS images of Deuterium-labelled lipid (CD_L_; 2145 cm^−1^), lipid (CH_2_; 2845 cm^−1^), Deuterium-labelled protein (CD_P_; 2175 cm^−1^) and protein (CH_3_; 2940 cm^−1^) channels of HeLa cells in different groups.
