# Supplementary figures and images for: Visualizing Cancer Cell Metabolic Dynamics Regulated With Aromatic Amino Acids Using DO-SRS and 2PEF Microscopy
Source: Front Mol Biosci. 2021 Dec 15;8:779702. doi: 10.3389/fmolb.2021.779702 (PMC8714916; doi:10.3389/fmolb.2021.779702)

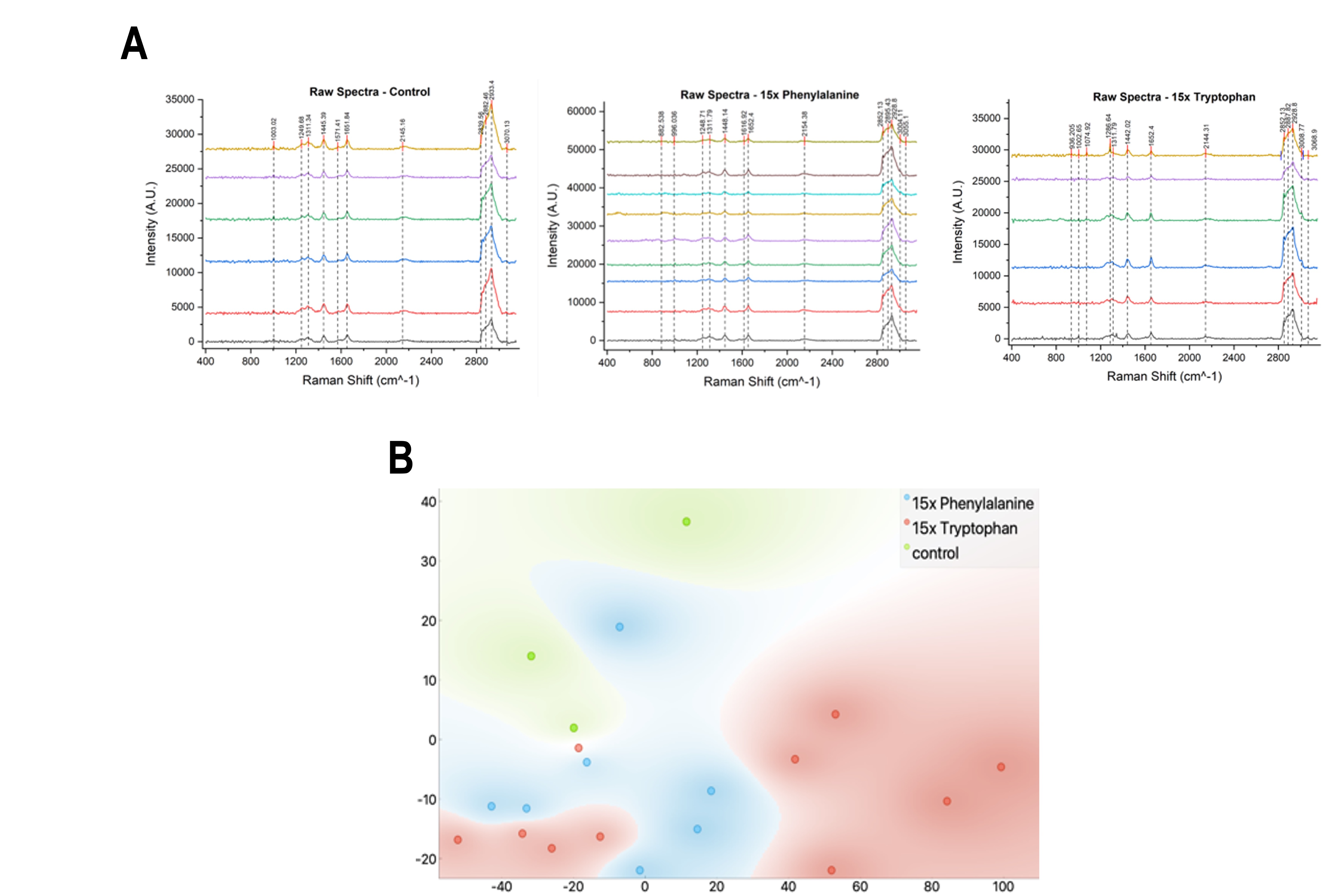

Supplement: Supplementary file 1 [file Image1.TIFF]

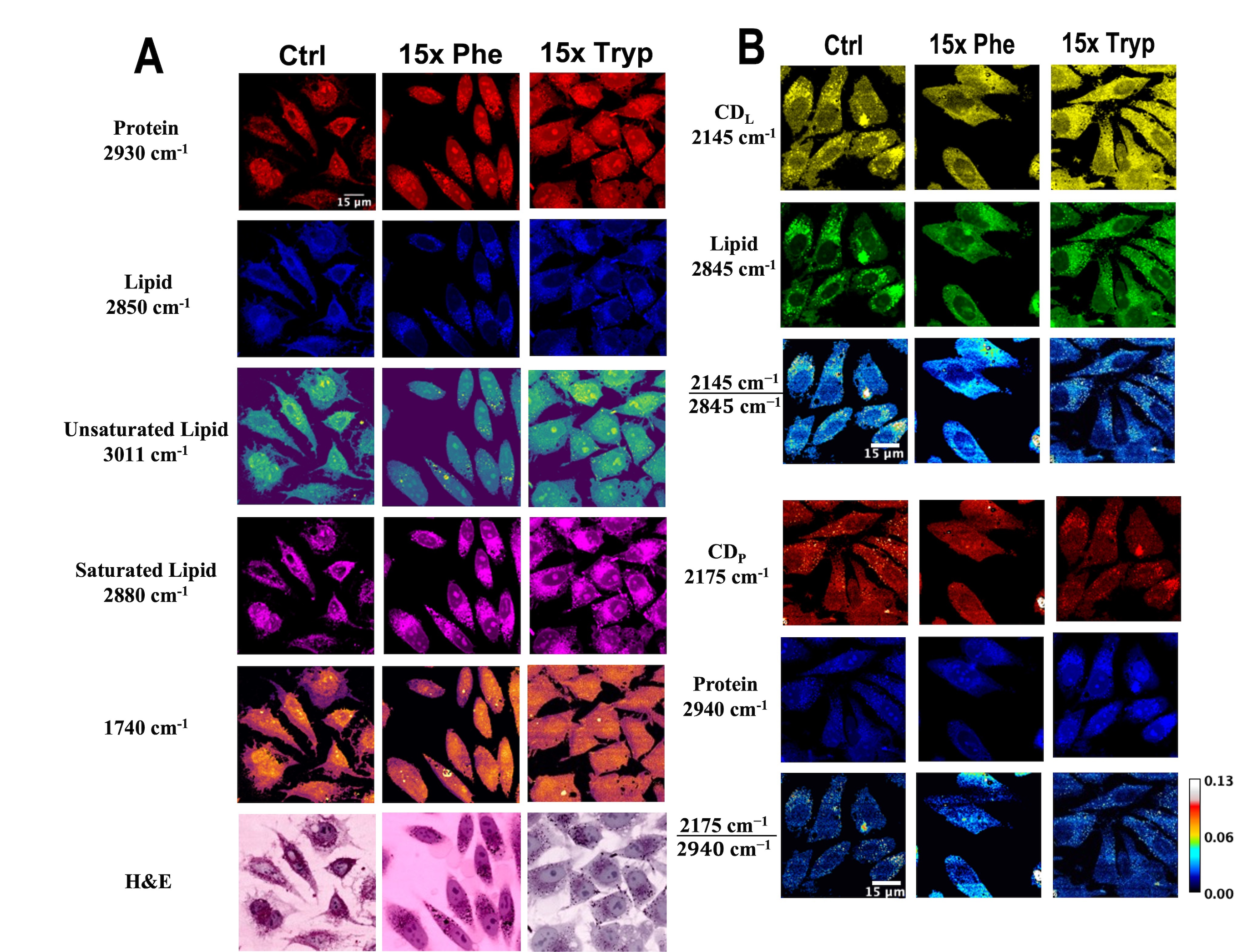

Supplement: Supplementary file 2 [file Image2.TIFF]
